# Supplementary material for: Genetic diversity and structure in two epiphytic orchids from the montane forests of southern Ecuador: The role of overcollection on Masdevallia rosea in comparison with the widespread Pleurothallis lilijae
Source: PLoS One. 2023 Sep 15;18(9):e0290604. doi: 10.1371/journal.pone.0290604 (PMC10503748; doi:10.1371/journal.pone.0290604)

**Supporting Information Files**

**Figure S1.** Number of genetic clusters in the studied population of *Pleurothallis lilijae* and *Masdevallia rosea* according to Structure. The log-likelihood of the data [lnP(X)] averaged over 10 consecutive Structure runs for K = 1 to 10, with error bars representing ± standard deviation for *P. lilijae* (a) and *M. rosea* (c). Evanno's ΔK statistic plotted against K for *P. lilijae* (b), and *M. rosea* (d).


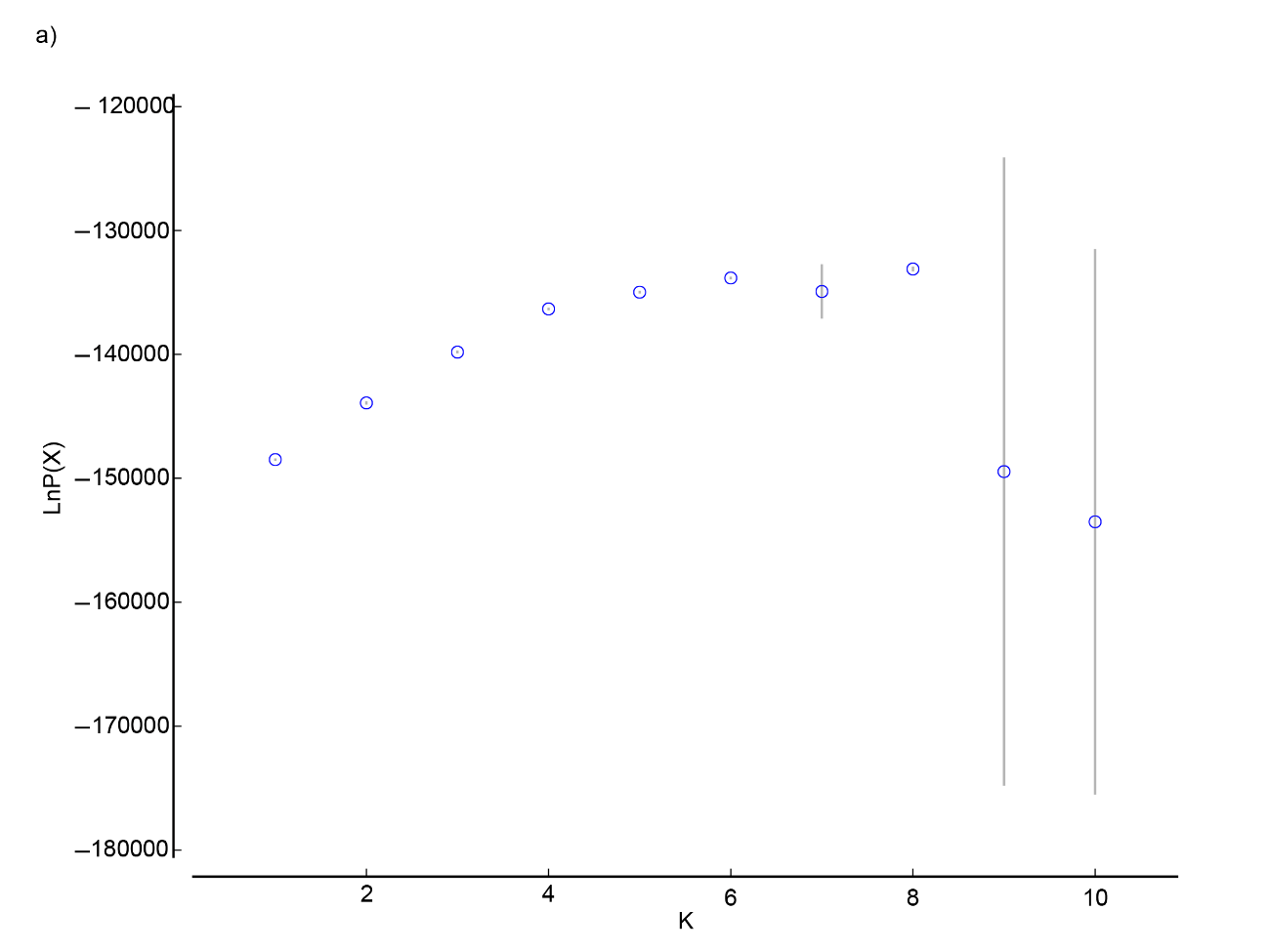


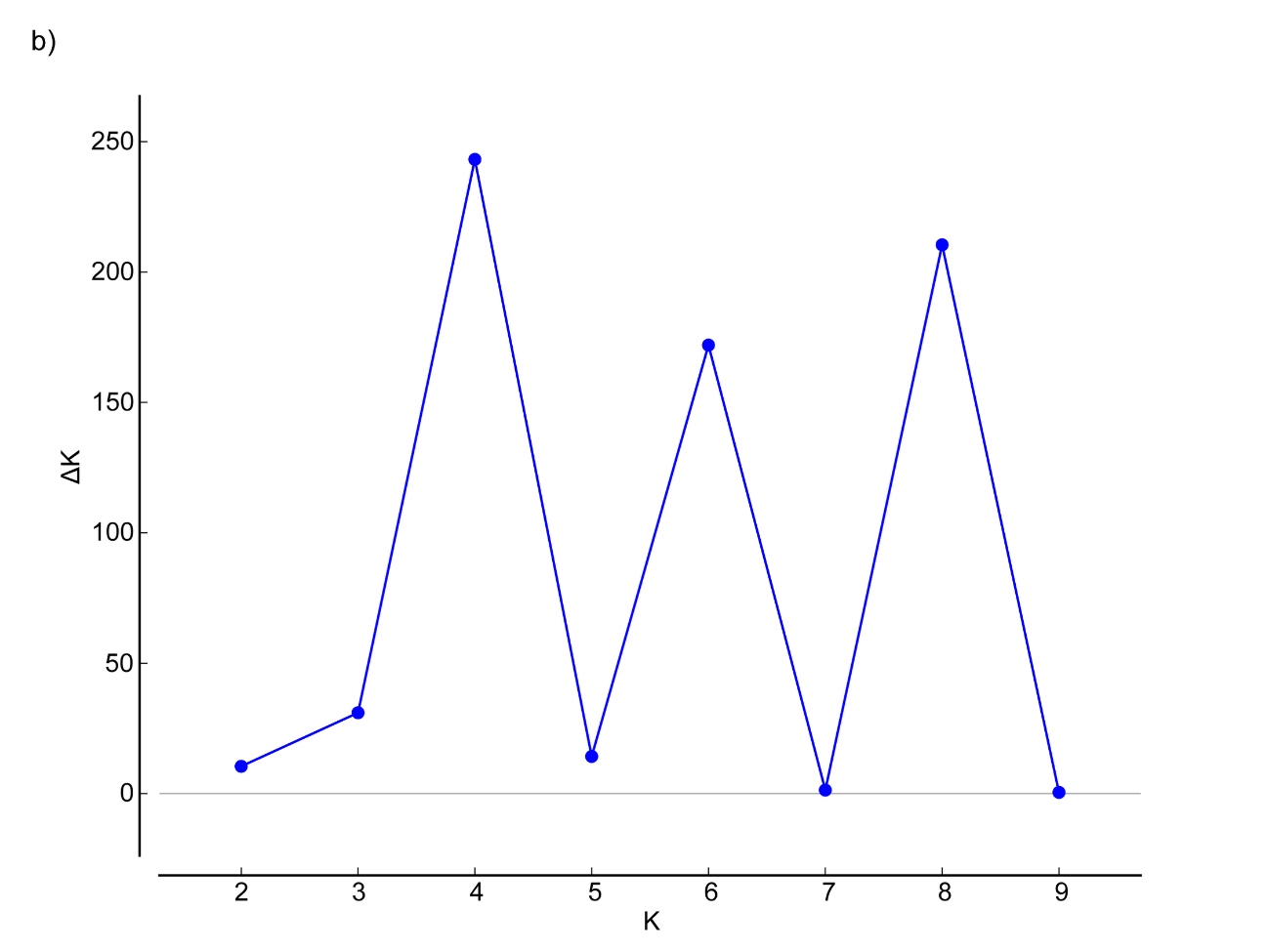


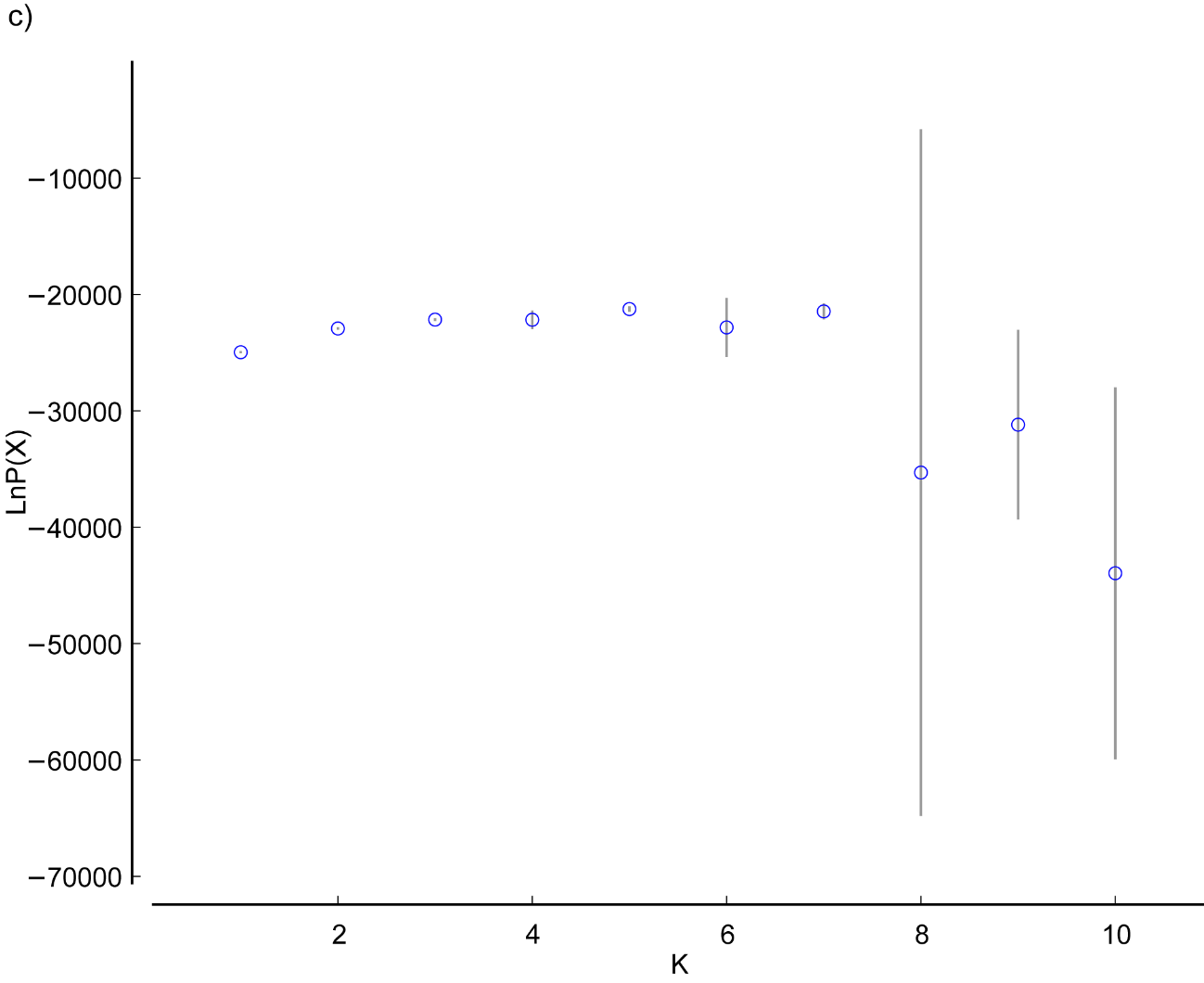


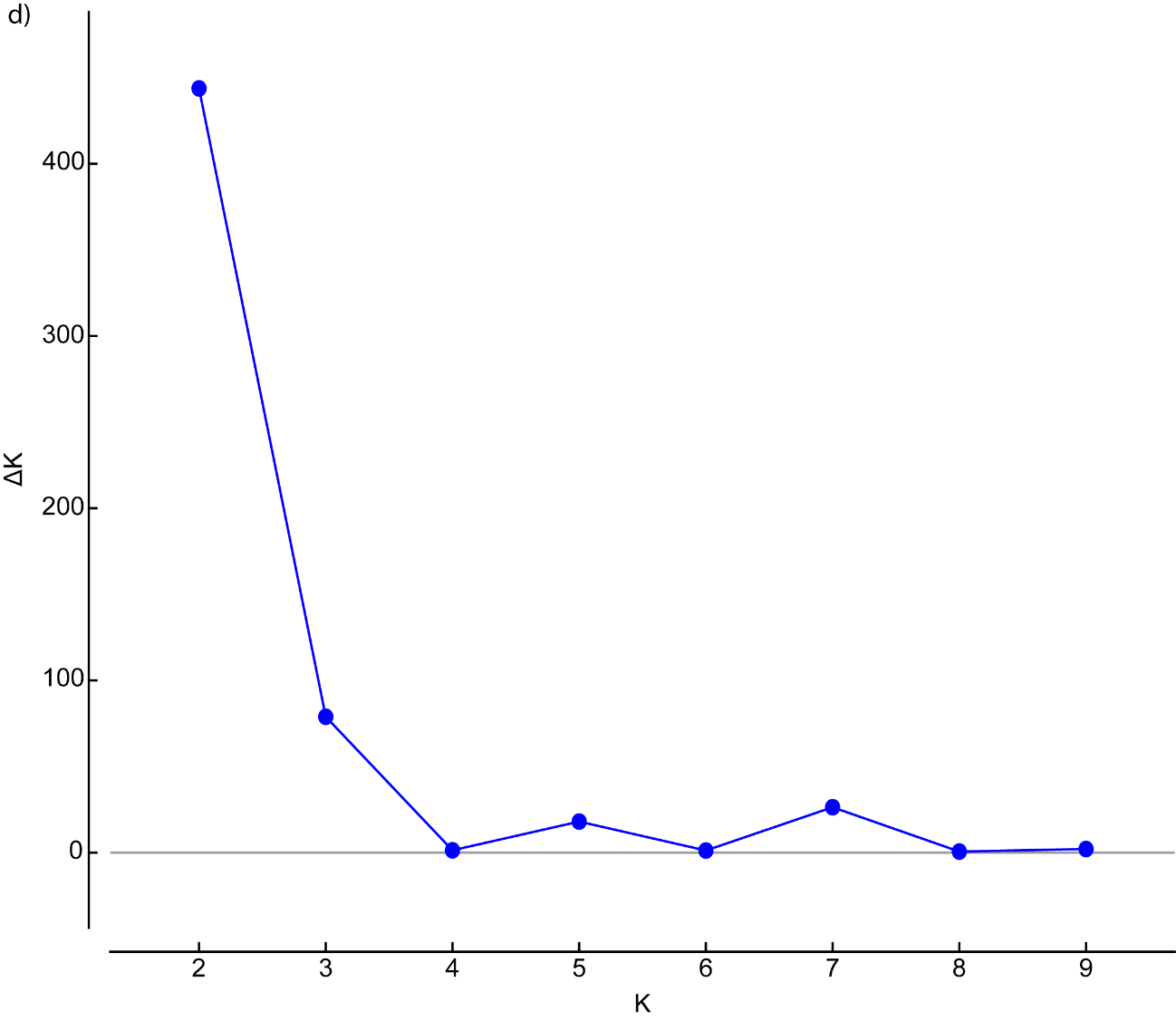

Supplement: S1 Fig — The log-likelihood of the data [lnP(X)] averaged over 10 consecutive Structure runs for K = 1 to 10, with error bars representing ± standard deviation for P. lilijae (a) and M. rosea (c). Evanno’s ΔK statistic plotted against K for P. lilijae (b), and M. rosea (d). (DOCX) [file pone.0290604.s001.docx]
