# Supplementary material for: Genetic diversity and structure in two epiphytic orchids from the montane forests of southern Ecuador: The role of overcollection on Masdevallia rosea in comparison with the widespread Pleurothallis lilijae
Source: PLoS One. 2023 Sep 15;18(9):e0290604. doi: 10.1371/journal.pone.0290604 (PMC10503748; doi:10.1371/journal.pone.0290604)
Supplement: Attachment — Submitted filename: PONE-D-22-26701reviewed.pdf [file pone.0290604.s002.pdf]

PLOS ONE

Understanding the genetic structure of two epiphytic orchids Pleurothallis lilijae and Masdevallia rosea in the montane forest, in southern Ecuador

--Manuscript Draft--

**Manuscript Number:**	PONE-D-22-26701
**Article Type:**	Research Article
**Full Title:**	Understanding the genetic structure of two epiphytic orchids Pleurothallis lilijae and Masdevallia rosea in the montane forest, in southern Ecuador
**Short Title:**	Genetic structure of two epiphytic orchid in montane forest in Ecuador
**Corresponding Author:**	María Lorena Riofrío Guamán, Ph.D<br>Universidad Técnica Particular de Loja<br>Loja, Loja ECUADOR
**Keywords:**	AFLPs; Pleurothallis lilijae; Masdevallia rosea; epiphytic orchid; genetic structure
**Abstract:**	In this study we tested the genetic structure of two epiphytic orchids located in a different altitudinal range, Pleurothallis lilijae with a high number of individuals distributed among six populations and Masdevallia rosea with few individuals in four populations and threatened by extraction for illegal trade. Individuals in their natural population were georeferenced and genotyped by AFLPS. This study reports a significant low genetic differentiation between the natural population in both species, genetic structure analysis reveals four genetic groups in P. lilijae while two in M. rosea ; a spatial genetic structure by altitudinal range was observed for P. lilijae , and a small distance for M. rosea . These results suggest different scenarios for the two species: possible hybridization events in P. lilijae and forest fragmentation processes could affect genetic population structure, while in M. rosea could be explained by anthropogenic pressure as a selection of individuals and their extraction for illegal trade. In situ and ex-situ conservation strategies must be applied to protect the genetic pool in this epiphytic orchid species.
**Order of Authors:**	María Lorena Riofrío Guamán, Ph.D
	Carlos James Naranjo, Master
	Alberto Eudaldo Mendoza, Engineer
**Additional Information:**	
**Question**	**Response**
**Financial Disclosure**<br><br>Enter a financial disclosure statement that describes the sources of funding for the work included in this submission. Review the submission guidelines for detailed requirements. View published research articles from *PLOS ONE* for specific examples.<br><br>This statement is required for submission and **will appear in the published article** if the submission is accepted. Please make sure it is accurate.	This research was supported by SENESCYT-ECUADOR and Universidad Técnica Particular de Loja

Powered by Editorial Manager® and ProduXion Manager® from Aries Systems Corporation

**Unfunded studies**<br>Enter: *The author(s) received no specific funding for this work.*<br><br>**Funded studies**<br>Enter a statement with the following details:<br>• Initials of the authors who received each award<br>• Grant numbers awarded to each author<br>• The full name of each funder<br>• URL of each funder website<br>• Did the sponsors or funders play any role in the study design, data collection and analysis, decision to publish, or preparation of the manuscript?<br>• **NO** - Include this sentence at the end of your statement: *The funders had no role in study design, data collection and analysis, decision to publish, or preparation of the manuscript.*<br>• **YES** - Specify the role(s) played.<br><br>* typeset	
**Competing Interests**<br><br>Use the instructions below to enter a competing interest statement for this submission. On behalf of all authors, disclose any competing interests that could be perceived to bias this work—acknowledging all financial support and any other relevant financial or non-financial competing interests.<br><br>This statement is required for submission and **will appear in the published article** if the submission is accepted. Please make sure it is accurate and that any funding sources listed in your Funding Information later in the submission form are also declared in your Financial Disclosure statement.<br><br>View published research articles from *PLOS ONE* for specific examples.	The authors have declared that no competing interest exist.

**NO authors have competing interests**<br><br>Enter: *The authors have declared that no competing interests exist.*<br><br>**Authors with competing interests**<br><br>Enter competing interest details beginning with this statement:<br><br>*I have read the journal's policy and the authors of this manuscript have the following competing interests: [insert competing interests here]*<br><br>* typeset	
**Ethics Statement**<br><br>Enter an ethics statement for this submission. This statement is required if the study involved:<br><br>• Human participants<br>• Human specimens or tissue<br>• Vertebrate animals or cephalopods<br>• Vertebrate embryos or tissues<br>• Field research<br><br>Write "N/A" if the submission does not require an ethics statement.<br><br>General guidance is provided below. Consult the submission guidelines for detailed instructions. **Make sure that all information entered here is included in the Methods section of the manuscript.**	MAE-DNB-CM-2015-0016<br>Ministerio del Ambiente-Ecuador

Powered by Editorial Manager® and ProduXion Manager® from Aries Systems Corporation

**Format for specific study types**<br><br>**Human Subject Research (involving human participants and/or tissue)**<br>• Give the name of the institutional review board or ethics committee that approved the study<br>• Include the approval number and/or a statement indicating approval of this research<br>• Indicate the form of consent obtained (written/oral) or the reason that consent was not obtained (e.g. the data were analyzed anonymously)<br><br>**Animal Research (involving vertebrate animals, embryos or tissues)**<br>• Provide the name of the Institutional Animal Care and Use Committee (IACUC) or other relevant ethics board that reviewed the study protocol, and indicate whether they approved this research or granted a formal waiver of ethical approval<br>• Include an approval number if one was obtained<br>• If the study involved *non-human primates*, add *additional details* about animal welfare and steps taken to ameliorate suffering<br>• If anesthesia, euthanasia, or any kind of animal sacrifice is part of the study, include briefly which substances and/or methods were applied<br><br>**Field Research**<br><br>Include the following details if this study involves the collection of plant, animal, or other materials from a natural setting:<br>• Field permit number<br>• Name of the institution or relevant body that granted permission	
**Data Availability**<br><br>Authors are required to make all data underlying the findings described fully available, without restriction, and from the time of publication. PLOS allows rare exceptions to address legal and ethical concerns. See the PLOS Data Policy and FAQ for detailed information.	No - some restrictions will apply

Powered by Editorial Manager® and ProduXion Manager® from Aries Systems Corporation

A Data Availability Statement describing where the data can be found is required at submission. Your answers to this question constitute the Data Availability Statement and **will be published in the article**, if accepted.<br><br>**Important:** Stating 'data available on request from the author' is not sufficient. If your data are only available upon request, select 'No' for the first question and explain your exceptional situation in the text box.<br><br>Do the authors confirm that all data underlying the findings described in their manuscript are fully available without restriction?	
**Describe where the data may be found in full sentences. If you are copying our sample text, replace any instances of XXX with the appropriate details.**<br><br>• If the data are **held or will be held in a public repository**, include URLs, accession numbers or DOIs. If this information will only be available after acceptance, indicate this by ticking the box below. For example: *All XXX files are available from the XXX database (accession number(s) XXX, XXX.).*<br>• If the data are all contained **within the manuscript and/or Supporting Information files**, enter the following: *All relevant data are within the manuscript and its Supporting Information files.*<br>• If neither of these applies but you are able to provide **details of access elsewhere**, with or without limitations, please do so. For example:<br><br>*Data cannot be shared publicly because of [XXX]. Data are available from the XXX Institutional Data Access / Ethics Committee (contact via XXX) for researchers who meet the criteria for access to confidential data.*<br><br>*The data underlying the results presented in the study are available from (include the name of the third party*	All relevant data are within the manuscript and its Supporting Information files.

and contact information or URL).<br>• This text is appropriate if the data are owned by a third party and authors do not have permission to share the data.<br><br>* typeset	
Additional data availability information:	Tick here if your circumstances are not covered by the questions above and you need the journal's help to make your data available.

Powered by Editorial Manager® and ProduXion Manager® from Aries Systems Corporation

Manuscript Click here to access/download;Manuscript;manuscript.docx

Understanding the genetic structure of two epiphytic orchids *Pleurothallis lilijae* and *Masdevallia rosea* in the montane forest, in southern Ecuador

María Riofrío[1*], Alberto Mendoza[1], Carlos Naranjo[1]

[1]Department of Biology Sciences, Universidad Técnica Particular de Loja, San Cayetano alto s/n, Loja, Ecuador

*Corresponding author

E-mail: mlriofrio@utpl.edu.ec (MR)

Abstract

In this study we tested the genetic structure of two epiphytic orchids located in a different altitudinal range, *Pleurothallis lilijae* with a high number of individuals distributed among six populations and *Masdevallia rosea* with few individuals in four populations and threatened by extraction for illegal trade. Individuals in their natural population were georeferenced and genotyped by AFLPS. This study reports a significant low genetic differentiation between the natural population in both species, genetic structure analysis reveals four genetic groups in *P. lilijae* while two in *M. rosea*; a spatial genetic structure by altitudinal range was observed for *P. lilijae*, and a small distance for *M. rosea*. These results suggest different scenarios for the two species: possible hybridization events in *P. lilijae* and forest fragmentation processes could affect genetic population structure, while in *M. rosea* could be explained by anthropogenic pressure as a selection of individuals and their extraction for illegal trade. In situ and ex-situ conservation strategies must be applied to protect the genetic pool in this epiphytic orchid species.

Introduction

Species develop abilities to adapt to changing environmental conditions, such as climate change and fragmentation processes due to deforestation, to maintain their populations and avoid extinction, therefore a good understanding of genetic structure is important for sustainable management of populations [1]. Genetic diversity in a spatial context that is Spatial Genetic Structure (SGS), contributes to an interpretation of mechanisms maintaining population dynamics and provides tools to understand genetic events, selective pressures, gene flow, and genetic drift [2-4].

In Ecuador 20% of plant diversity corresponds to orchids [5-7]; more than 60% of orchids are epiphytes and characteristic in montane forests and montane cloud forests [5,8]. Despite this high diversity, orchids are affected by several threats, mainly deforestation, as they are dependent on the forest that supports them [9]. For Ecuador deforestation occupy a high rate [10,11], as a consequence of land clearing to increase the agricultural frontier, mining, and new infrastructure [12] which have caused the fragmentation of the forest [13], leaving small remnants and threatening the survival of the species population [14], only fragmented forest are presented, with natural variation in species, structure, composition, caused by habitat heterogeneity [15]. In addition to this, orchids face other anthropogenic threats of direct impact such as the extraction of orchids destined for illegal trade [6] that has led to the extinction of the orchid population, or indirect impact such as climate change [14]. Therefore, the focus on studies that allow knowing the diversity and genetic structure of orchid populations is necessary to understand the effects that anthropogenic factors have caused in natural populations and to be able to propose viable conservation strategies.

This study assessed the genetic structure of two epiphytic orchids with populations located at two altitude ranges; one is *Pleurothallis lilijae* of large distribution and abundance, the other *Masdevallia rosea* of restricted distribution and historically has been extracted for their population for trade, to infer genetic structure and understand the factors that could be affecting them. We consider the following questions: 1) What is the genetic status of both species? 2) Are there genetic structures in populations of each species? 3) Is genetic structure affected by anthropogenic factors in *P. lilijae* and *M. rosea* populations?

Materials and Methods

Study site and sampling

The study site was located on “Cordillera Real” in the Andes of southern Ecuador, at Podocarpus National Park, with 3 sites (Fig1). Site 1, Cajanuma: it is located at an altitude of 3000 m a.s.l. approximately and has an annual average temperature of 9.4 ° C and relative humidity of 91%. It is a forest classified as high montane evergreen forest [16] with sclerophyllous to subsclerophyllous and lauroid forests, generally dense and with two woody strata, abundant epiphytes, and mosses. Site 2, located at San Francisco Biological Reserve located at an altitude of 2000 m above sea level approximately. It is located northwest of Cerro El Consuelo, on the banks of the San Francisco River. It has an average annual temperature of 15°C and average relative humidity of 96.3%. It is dominated by a montane evergreen forest with a multi-stratified evergreen forest, with bryophytes and epiphytes present in abundance [16]. Site 3, El Tiro located at 2800 m a.s.l. approximately at the north limit of Podocarpus National Park, is crossed by the main highway from Loja to Zamora and the old highway to Zamora (not currently enabled). El Tiro is about 10 km from Loja, on the crest of the mountain range, on the political border between the provinces of Loja and Zamora Chinchipe. The ecosystem corresponds to a sub-paramo of woody, shrubby and diverse vegetation, and low-stature forests. It is a forest classified as high montane evergreen forest [16].

Two orchid species were located and evaluated in the study area: *Pleurothallis lilijae* Foldats and *Masdevallia rosea* (Lindl.) than are photosynthetic epiphytic perennial orchids), this orchids species below subtribe Pleurothallidinae, with a ramicauls bearing one leave), with lack pseudobulb [17]. *Pleurothallis lilijae* is a specie with a larger distribution range and abundance in contrast to *Masdevallia rosea* with a more restricted distribution and conditioned by illegal extraction that is done for trade.

Orchid populations were found in sites with different altitude ranges, a high range of 2826–2917 m a.s.l. (site 1), a medium altitude range of 1988–2266 m a.s.l. (site 2), and a third site with a medium-high altitude range of 2686-2747 m a.s.l. (site 3). For each species, the population was located in sites one and two for *P. lilijae*, and sites one and three for *M. rosea*. In every orchid population, each individual with flowers was located, counted, and marked. One orchid by phorophyte was sampled for DNA extraction. Orchid phorophyte was georeferenced.

Fig 1. Distribution map of six populations of *P. lilijae* and three populations of *M. rosea*. The red circle represents *P. lilijae* populations and the yellow circle represents *M. rosea* populations.

Sampling for genetic analyses and DNA extraction

Young leaf samples of adult individuals of *P. lilijae* and *M. rosea* present in phorophytes were collected for DNA analysis. Leaf material was dried in silica gel and stored at room temperature until DNA extraction.

Total DNA was extracted from 30 mg of dried leaf material using Plant Mini Kit (Quiagen, Hilden, Germany). After extraction, DNA concentration was estimated using a fluorospectrometer (NanoDrop) and agarose gel (25). DNA samples were stored at -20 ºC until use.

Amplified Fragment Length Polymorphism (AFLP) procedure

AFLP reactions were performed following the procedure of Vos et al. [18]with minor modifications. Genomic DNA (approximately 500 ng) was restricted with 0.1 units of *Mse*I (New England BioLabs, Ipswich, Massachusetts, USA) and 0.5 units of *Eco*RI (Takara Bio Inc., Otsu, Japan) endonucleases, and ligated to MseI and EcoRI adapters with 6 units of T4

DNA-ligase (Takara Bio Inc.). Samples were incubated in a thermocycler for 3 h at 37º C and 1 h at 17º C.

Preselective amplification was performed using the primers that complement the MseI and EcoRI adaptors plus one additional nucleotide i.e. MseI+C (5’ GAT GAG TCC TGA GTA AC 3') and EcoRI+A (5’-GAC TGC GTA CCA ATT CA-3’). PCR reactions were conducted in 6.25 µl reaction volume containing 1.25 µl of 10-fold diluted restriction-ligation product, 1X buffer (GeneAmp 10X PCR Buffer II, Applied Biosystems, California, USA), 1.5 mM $MgCl_2$, 0.2 mM dNTPs, 0.2 µM of each primer, and 0.5 units of Taq polymerase (AmpliTaq DNA Polymerase, Applied Biosystems). The thermocycler program used for amplifications was as follows: 72 ºC for 2 min first, then 30 cycles at 94 ºC for 30 s, 56 ºC for 30 s, and 72 ºC for 2 min, with a final extension at 72 ºC for 10 min. The quality of undiluted preselective and restricted/ligation products was tested on 1% (w/v) agarose gels.

Selective amplifications were conducted in a reaction volume of 6.25 µl containing 1.25 µl of 5-fold diluted preselective product, 1X buffer (GeneAmp 10X PCR Buffer II, Applied Biosystems), 1.5 mM $MgCl_2$, 0.8 mM dNTPs, 0.08 µM of EcoRI fluorescent primer (Applied Biosystems), 0.2 µM of MseI primer (Bonsai Tech), and 0.5 U of Taq polymerase (AmpliTaq Gold DNA Polymerase, Applied Biosystems). PCR conditions were: 95 ºC for 2 min first, then 13 cycles at 94 ºC for 30 s, 65 ºC for 1 min (with a decreasing gradient of 0.7 ºC every cycle), and 72 ºC for 2 min; and 24 cycles at 94 ºC for 30 s, 56 ºC for 1 min, and 72 ºC for 2 min, with a final extension at 72 ºC for 10 min.

Four primer combinations producing clear bands were used for selective amplification: EcoRI AAC NED – MseI CAC, EcoRI AGG VIC – MseI CTC, EcoRI AGA FAM – MseI CTA, y EcoRI AGA FAM – MseI CAC. The selection was based on preliminary screening of 10 primer combinations in six individuals (including all populations).

AFLP fragments were separated on an ABI 3500 sequencer (Applied Biosystems). A gene scan 500 Liz-labelled sizes standard (Applied Biosystems) was injected with each AFLP sample to allow sizing of the DNA fragments.

Data analysis

For each species, AFLPs profiles were imported into GeneMarker v.3.0.0 (Softgenetics, LLC) and scored manually. All bands between 80 and 500 bp were scored as present (1) or absent (0) excluding those that could not be decisively assigned. Monomorphic and polymorphic fragments were included in the analysis.

Detection of genetic structure

As a preliminary step for each species a principal coordinates analysis (PCoA) was implemented to provide a visual representation of the genetic distance relationships among individuals of *P. lilijae*. Following the suggestions of Bonin et al. [19], a simple matching coefficient was used to calculate the distance matrix.

Next, population structure was inferred by applying model-based clustering methods. Unlike distance-based clustering, these methods use a model to identify genetic groups from the full set of genotype data and to assign probabilistically individuals (or a fraction of their genomes) to that group representing the best fit for the various patterns found. We used the approach implemented in Structure v. 2.3.4, which involves Bayesian inference and parameter estimation through Markov Chain Monte Carlo (MCMC) in the modeling process [20,21]. For analysis with Structure, we used the *admixture model* (which assumes individuals may have mixed ancestry) with *correlated allele frequencies* [22] and set the number of groups (K) from 1 to 10. For each value of K, 5 independent runs were carried out with a burn-in period of 10,000 followed by 300,000 MCMC iterations. The optimal number

of groups was determined by estimating the log-likelihood of the data for each *K* [ln(P(X|K)] as suggested by Pritchard et al. [20], and the ΔK statistic proposed by Evanno et al. [23] which is based on the rate of change in the log-likelihood of data between successive *K* values. Afterward, the software CLUMPP v. 1.1.2 [24] was used, employing the *FullSearch* algorithm, to find the optimal alignment of the 5 independent replicate cluster analyses, and to compute the mean membership coefficient matrix (Q-matrix).

Detection of spatial genetic structure

The spatial genetic structure was assessed using the spatial autocorrelation method proposed by Smouse & Peakall [25], which allows simultaneously assessing the signal generated by multiple loci. Briefly, this approach calculates an autocorrelation coefficient (*r*) between genetic and geographic distances for all pairs of individuals within user-specified distance classes. Under isolation by distance, geographically close individuals are expected to be more genetically similar amongst them than to other individuals occurring at greater distances. Therefore, $r > 0$ values are expected for short-distance lags and $r < 0$ for long-distance lags.

Genetic distances between individuals were calculated using the method of Huff et al. [26]. For distance classes, we considered a classical rule of thumb where only pairs of points separated by less than half the maximum distance observed were included [27]. For *P. lilijae* was 9599 m and for *M. rosea* was 7224 m. Distance classes were defined at intervals of 300 m for both species in whole analyses. When considering maximum distance in population by altitudinal range for distance class, intervals were 100m.[28]Distance between individuals was calculated from x, y, and z coordinates. Each value of *r* was tested for significant deviations from the expected value under the null hypothesis of no spatial genetic structure by a permutation test in which the whole AFLP phenotypes were randomly

shuffled among the spatial positions occupied and *r*-values recalculated each time (up to a maximum of 999 times) [see Smouse and Peakall (1999) and Smouse et al. (2008) for details]. The significance of autocorrelation (*r)* was constructed with one-tailed probability values calculated. The significance of *r* was also tested by generating bootstrap 95% confidence intervals around the mean value of *r*. Bootstrap values were obtained by sampling, with replacement, pairs of comparisons within a given distance class [28]. Bootstrap resampling was performed 999 times and the significance of *r* was inferred when the 95% confidence interval did not contain the zero value. The significance of the entire correlogram was tested using the heterogeneity test proposed by Smouse et al. [29]. All analyses were implemented in GenAlEx v.6.5 [30].

Results

For *P.* lilijae 1018 individuals were located in six populations distributed, three at high altitude range-Cajanuma (site 1) and three at medium altitude range-San Francisco (site2). For *M. rosea* 107 individuals were located, distributed in four population, two at high altitude range-Cajanuma (site 1) and two a medium-high altitude-El Tiro (site 3). AFLP analysis was successful in 328 individuals for *P. lilijae* and provide a total of 810 bands. Also, the analysis was successful in 95 individuals of *M. rosea*, with 664 bands. For both species, bands were between 80 and 500 bp. 100% of the studied loci were polymorphic.

Population genetic structure

The scatter plot for the first and second axis of the PCoA analysis show two genetic groups for *P. lilijae* (fig. 2a), with 8.74% of the variance explained in the first two axes (4.68% for PCoA1, and 4.06% for PCoA2). This PCoA indicates a grouping for the population of the high-altitude range and another for the population of the medium-altitude range. For *M.*

rosea PCoA analysis indicates two genetic groups (fig. 2b). The first two principal coordinates accounted for 16.63% of the total variance (11.31% PCoA1 and 5.32% PCoA2), similar to *P. lilijae* genetic grouping is visualized in the two altitude ranges where the populations were located. For *M. rosea*, a population located in the medium-high altitude range, from site 2 “El Tiro” is more grouped regarding the others.

Fig. 2. Bidimensional plot of PCoA obtained of four combinations of AFLPs a) for *P. lilijae* and b) for *M. rosea*.

The Bayesian clustering analysis conducted by Structure identified four genetic groups for *P. lilijae*, in Fig 3a-b the first value lnP(X) at which the curve reaches the plateau (-1333835.62) and the highest ΔK value (243.195) were found for K = 4 (although highest values were found for K6 and K8), indicating that the genetic structure produced by dividing all genotypes into four structured groups is the most likely; in high altitudinal range two genetic groups had admixture coefficients over 0.8 (30 individuals), and in medium altitudinal range two different genetic groups had high admixture coefficient (58 individuals) (fig4a). For *M. rosea* in Fig 3b-c, see that lnP(X) (-22917.44) and ΔK value (443.747) suggest the number of clusters present in the data to be two; with an admixture coefficient over 0,8 for 29 individuals in high altitudinal range distributed in two genetic groups, while for the medium-high altitude range exists 55 individuals with admixture coefficient over 0,8 represented in only one genetic groups (fig 4b). As in PCoA, two genetic groups were present in high and medium-high altitude range.

Fig. 3. Number of genetic clusters in the studied population of *P. lilijae* and *M. rosea* according to Structure. The log-likelihood of the data [lnP(X)] averaged over 10 consecutive Structure runs for K = 1 to 10, with error bars representing ± standard deviation for *P. lilijae*

(a) and *M. rosea* (c). Evanno's ΔK statistic plotted against K for *P. lilijae* (b), and *M. rosea* (d).

Fig. 4. Bar-graph representing the admixture percentages for each individual *P. lilijae* and *M. rosea* according to the admixture model with correlated alleles implemented in Structure for K=4 in *P. lilijae* (a) and K=2 in *M. rosea* (b). The names at the bottom of the chart represent the population in each altitude range (Cajanuma: high altitude range, San Francisco: medium-altitude range, and El Tiro: medium-high altitude range), and the black vertical/horizontal lines delimitate the boundaries of each population.

For the whole population in each species, genetic differentiation according to the analysis of molecular variance (AMOVA) indicates that 6% of the genetic variance can be attributed to differences between populations for *P. lilijae* ($\phi_{ST} = 0.057$, $P < 0.001$), while low level of genetic differentiation was showed for *M. rosea* ($\phi_{ST} = 0.011$, $P < 0.003$) with 1%, and similar values of Shannon s information index and expected heterozygosity (see S1 table 1). When analyzed population by altitude ranges for each species, the values varied; for *P. lilijae* in high altitude range molecular variance among population was 2% ($\phi_{ST} = 0.018$, $P < 0.001$), and in medium altitude range was 3% ($\phi_{ST} = 0.026$, $P < 0.001$); for *M. rosea* in high altitude range molecular variance among populations was 5% ($\phi_{ST} = 0.052$, $P < 0.006$), and in medium-high altitude range was 12% ($\phi_{ST} = 0.119$, $P < 0.001$).

Spatial genetic structure

For each species in a first analysis, all individuals were analyzed no genetic spatial autocorrelation was observed neither in *P. lilijae* (ω=112,579, *P*<0.119) nor in *M. rosea* (ω=40.06, *P*<0.447), for whole individuals, correlogram is not significant (Fig 5a-b). But when we considered sampling size according to distance between population in each

altitudinal range, a positive spatial autocorrelation was observed for *P. lilijae* ($\omega=244.169$, $P<0.004$), significant positive autocorrelation was obtained in the first third distance classes (100 m, $r=0.049$, $P<0.001$; 200m, $r=0.031$, $P<0.001$; 300 m, $r=0.037$, $P<0.001$), in distance classes between 600 until 900 (600 m, $r=0.03$, $P<0.001$; 700m, $r=0.031$, $P<0.001$; 800 m, $r=0.032$, $P<0.001$; 900 m, $r=0.028$, $P<0.001$), 1400 until 1500 (1400 m, $r=0.031$, $P<0.001$; 1500 m, $r=0.017$, $P<0.001$), 2800 until 3000 (2800 m, $r=0.022$, $P<0.001$; 2900m, $r=0.025$, $P<0.001$; 3000 m, $r=0.025$, $P<0.002$), 3200 until 3400 (3200 m, $r=0.030$, $P<0.001$; 3300m, $r=0.023$, $P<0.001$; 3400 m, $r=0.029$, $P<0.001$), and a first x-intercept of r at 2611.14m, evidencing the presence of spatial genetic structure within population at this distance class (fig. 5c). For *M. rosea* significate autocorrelation was observed in whole individuals when used distance class according to *intrapopulation* distance ($\omega=49.846$, $P<0.001$), with significant positive autocorrelation in the four distance classes (50 m, $r=0.097$, $P<0.001$; 100m, $r=0.077$, $P<0.001$; 150 m, $r=0.069$, $P<0.001$, 200 m, $r=0.079$, $P<0.021$) (Fig. 5d).

Fig. 5. Spatial genetic structure of *P. lilijae* (a-c) and *M. rosea* (b) showing the genetic correlation coefficient (r) as a function of geographical distance. Autocorrelograms are based on geographical distance classes of 9500 m (a), 7200 m (b), 4000 m (c), and 300m (d). Dashed lines represent upper and lower 95% confidence limits, whereas the solid blue line represents r (spatial autocorrelation coefficient).

Discussion

This study analyzed the genetic structure of two epiphytic orchid populations in three sites that correspond to altitude ranges (high, medium, medium-high) in a montane forest contrasting with anthropogenic factors that could affect these populations: *P. lilijae* with a

large number of individuals, while *M. rosea* with few individuals in their population and threatened by the extraction of the resource for trade.

Population genetic structure

The presence of the same four genetic groups in six populations of *P. lilijae* both from the high and medium altitude range (with a separation distance of approximately 18940 m) revealed the presence of high gene flow and admixture in populations, a possible answer is that there are hybridization processes with other sympatric species of *Pleurothallis* in high altitudinal range, in other epiphytic orchid species, hybridization events are presented in populations between sympatric orchids [31-33] contributing to genetic variation and, due to long-distance seed dispersal events, possibly mediated by wind [34] these genotypes distribute in the whole population in the two altitude ranges, additional studies are necessary to verify possible hybridization events with other sympatric species of *Pleurothallis*.

On the other hand, the lack of spatial genetic structure when taking (when half sampling size was considered [27]) as a whole individual could suggest that the species has a long-distance seed dispersal mechanism [27]. When we consider sampling size as maximum distance in each altitude range we found significant correlograms obtained for distance class 100 m, this positive spatial genetic structure shows an x-intercept of *r* at 2611 m, being able to consider the size of the genetic patch and the scale in which the pattern occurs [2,28], this distance would be within the maximum distances ranges of the populations in each site, this result is consistent with PCoA results for *P. lilijae* where two groups were observed corresponding to each altitude range.

In *M. rosea* two different genetic groups were found in the Bayesian clustering approach Structure and PCoA, for the population located in the medium-high altitude range, all the

individuals had percentages of belonging to a genetic group above 80%, indicating that these populations are possibly subject to anthropogenic pressures of selection of individuals for their extraction, and therefore only one of the genotypes is predominating, in the same way possibly the genotype that is found in a lower proportion is the one that corresponds to floral characteristics that are more attractive for trade; these populations have closer access to the highway, which would cause people to easily access the population and extract individuals for trade. While in the populations of the high-altitude range, the same genetic groups are found distributed in a similar proportion in individuals with more than 80% of admixture, this site is farther from the road and its access is more limited.

Is genetic structure affected by anthropogenic factors?

There are not many works that relate the genetic results between populations with the anthropogenic effects such as selective extraction for the trade of species, therefore the results that we can find in this article give us guidelines in the case of *M. rosea* so that the measures of protection and conservation for this species and other orchid species are intensified since it can be evidenced that in the populations where there is easy access, the individuals that remain present a prevalence of one genotype over the other.

Implications for the conservation of this species should be aimed at maintaining the gene flow between the populations and reducing the extraction of adult individuals for illegal trade, through the sensitization of local populations about the current status of the species, as well as the approach of new measures or regulations by the corresponding government entities that protect the populations in situ and avoid extractivism for illegal trade. On the other hand both *P. lilijae* and *M. rosea, in situ* and *ex situ* conservation strategies should be considered. *In situ* conservation allows them to maintain their genetic pool and the

conditions of their habitat [35], in addition to the interaction that these plants have with other organisms such as mycorrhizal fungi [36] or pollinators [37,38], then this conservation strategy will permit maintenance of the habitat for this group of epiphytic orchids dependent on the conditions of the environment; *ex situ* conservation strategies that allow the storage of seeds from all populations could help maintain the genetic diversity of these species for future reintroduction plans to increase population size in places where there is greater extractivism.

Acknowledgments

This research was supported by the SENESCYT-ECUADOR and Universidad Técnica Particular de Loja. We thank Daniela Arias for her help in the laboratory, and M. Naranjo and A. Poma for their help in part of the data analysis.

Author Contributions

Conceptualization: Lorena Riofrío

Data Curation: Alberto Mendoza

Formal Analysis: Lorena Riofrío, Alberto Mendoza, Carlos Naranjo

Funding Acquisition: Secretaria de Educación Superior, Ciencia, Tecnología e Innovación

Investigation: Lorena Riofrío

Methodology: Carlos Naranjo, Lorena Riofrío, Alberto Mendoza

Project Administration: Lorena Riofrío, Carlos Naranjo

Writing-Original Draft Preparation: Lorena Riofrío

Writing-Review & Editing: Lorena Riofrío

References

1. Machon N, Bardin P, Mazer SJ, Moret J, Godelle B, Austerlitz F. Relationship between genetic structure and seed and pollen dispersal in the endangered orchid *Spiranthes spiralis* New Phytologist. 2003;157: 677-687.

2. Escudero A, Iriondo J, Torres ME. Spatial analysis of genetic diversity as a tool for plant conservation. Biological Conservation. 2002;113: 351-365.

3. Jacquemyn H, Wiegand T, Vandepitte K, Brys R, Roldán-Ruiz I, Honnay O. Multigenerational analysis of spatial structure in the terrestrial, food-deceptive orchid *Orchis mascula* Journal of Ecology. 2009;97: 206-216.

4. Vekemans X, Hardy OJ. New insights from fine-scale spatial genetic structure analyses in plant populations. Molecular Ecology. 2004;13: 921-935.

5. Dodson C. Native Ecuadorian Orchids. 4th ed. Quito: Colina; 2003.

6. Endara L. Orchidaceae. In: León-Yañez S, Valencia R, Pitman N, Endara L, Ulloa C, Navarrete H, editors. Libro rojo de plantas endémicas del Ecuador. Quito: Publicaciones del Herbario QCA. Pontificia Universidad Católica del Ecuador; 2011. pp. 441-702.

7. Valencia R, Pitman N, León-Yañez S JP. Libro Rojo de las plantas endêmicas del Ecuador. 1st ed. Quito-Ecuador: Publicaciones del Herbario QCA. Pontifícia Universidad Católica del Ecuador; 2000.

8. Gravendeel B, Smithson A, Slik F, Schuiteman A. Epiphytism and pollinator specialization: drivers for orchid diversity? Philosophical transactions of the Royal Society of London. Series B: Biological sciences. 2004;359: 1523-1535.

9. Riofrío ML, Naranjo CJ, Iriondo JM, Torres ME. Spatial structure of *Pleurothallis, Masdevallia, Lepanthes* and *Epidendrum* epiphytic orchids in a fragment of montane cloud forest in south Ecuador. Lankesteriana. 2007;7: 102-106.

10. Tapia-Armijos M, Homeier J, Espinosa CI, Leuschner C, de la Cruz M. Deforestation and Forest Fragmentation in South Ecuador since the 1970s – Losing a Hotspot of Biodiversity. PLOS ONE. 2015;10: e0133701.

11. FAO. State of the world's forests. 1st ed. Rome: Food and Agriculture Organization of the United Nations; 2005.

12. Mosandl R, Gunter S, Stimm B, Weber M. Ecuador suffers the highest deforestation rate in south America. In: Beck E, Bendix J, Kottke I, Makeschin F, Mosandl R, editors. Gradients in a tropical mountain ecosystem of Ecuador. Germany: Springer-Verlag Berlin Heidelberg; 2008. pp. 37-40.

13. Santos T, Tellería J. Pérdida y fragmentación del hábitat: efecto sobre la conservación de las especies. Ecosistemas. 2006;15: 3-12.

14. Richter M, Diertl K, Emck P, Peters T, Beck E. Reasons for an outstanding plant diversity in the tropical Andes of Southern Ecuador. Landscape Online. 2009;12: 1-35.

15. Williams-Linera G. Tree species richness complementarity, disturbance and fragmentation in a Mexican tropical montane cloud forest. Biodiversity and Conservation. 2002;11: 1825-1843.

16. Báez S, Salgado S, Santiana J, Cuesta F, Peralvo M, Galeas R, et al. Propuesta Metodológica para la Representación Cartográfica de los Ecosistemas del Ecuador Continental. MAE. 2010.

17. Higgins WE, Williams NH. Pleurothallidinae: how many genera. . 2009: 425-430.

18. Vos P, Hogers R, Bleeker M, Reijans M, Van de Lee T, Hornes M, et al. AFLP: a new technique for DNA fingerprinting. Nucleic Acids Research. 1995;23: 4407.

19. Bonin A, Ehrich D, Manel S. Statistical analysis of amplified fragment length polymorphism data: a toolbox for molecular ecologists and evolutionists. Molecular Ecology. 2007;16: 3737-3758.

20. Pritchard J, Stephens M, Donnelly P. Inference of population structure using multilocus genotype data. Genetics. 2000;155: 945-959.

21. Falush D, Stephens M, Pritchard J. Inference of population structure using multilocus genotype data: dominant markers and null alleles. Molecular Ecology Notes. 2007;7: 574-578.

22. Falush D, Stephens M, Pritchard JK. Inference of population structure using multilocus genotype data: linked loci and correlated allele frequencies. Genetics. 2003;164: 1567-1587.

23. Evanno G, Regnaut S, Goudet J. Detecting the number of clusters of individuals using the software structure: a simulation study. Molecular Ecology. 2005;14: 2611-2620.

24. Jakobsson M, Rosenberg NA. CLUMPP: a cluster matching and permutation program for dealing with label switching and multimodality in analysis of population structure. Bioinformatics. 2007;23: 1801-1806.

25. Smouse PE, Peakall R. Spatial autocorrelation analysis of individual multiallele and multilocus genetic structure. Heredity. 1999;82: 561-573.

26. Huff DR, Peakall R, Smouse PE. RAPD variation within and among populations of outcrossing buffalograss [*Buchloë dactyloides* (Nutt.) Engelm]. Theoretical and Applied Genetics. 1993;86: 927-934.

27. Le Corre V, Roussel G, Zanetto A, Kremer A. Geographical structure of gene diversity in Quercus petraea (Matt.) Liebl. III. Patterns of variation identified by geostatistical analyses. Heredity. 1998;80: 464-473.

28. Peakall R, Ruibal M, Lindenmayer DB. Spatial autocorrelation analysis offers new insights into gene flow in the Australian bush rat, *Rattus fuscipes*. Evolution. 2003;57: 1182-1195.

29. Smouse P, Peakall R, González E. A heterogeneity test for fine-scale genetic structure. Molecular Ecology. 2008;17: 3389-3400.

30. Peakall R, Smouse P. GenAlEx 6.5: genetic analysis in Excel. Population genetic software for teaching and research—an update. Bioinformatics. 2012;28: 2537-2539.

31. Torres E, Riofrío M, Iriondo JM. Complex fine-scale spatial genetic structure in Epidendrum rhopalostele: an epiphytic orchid. Heredity. 2019;122: 458-467. doi: 10.1038/s41437-018-0139-1.

32. Sujii PS, Cozzolino S, Pinheiro F. Hybridization and geographic distribution shapes the spatial genetic structure of two co-occurring orchid species. Heredity. 2019;123: 458-469. doi: 10.1038/s41437-019-0254-7.

33. Marques I, Draper D, Riofrío ML, Naranjo C. Multiple hybridization events, polyploidy and low postmating isolation entangle the evolution of neotropical species of *Epidendrum* (Orchidaceae). BMC Evolutionary Biology. 2014;14: 20.

34. Arditti J, Ghani AK. Numerical and physical properties of orchid seeds and their biological implications. New Phytologist. 2000;145: 367-421.

35. Zhao Y, Tang M, Bi Y. Nuclear genetic diversity and population structure of a vulnerable and endemic orchid (Cymbidium tortisepalum) in Northwestern Yunnan, China. Scientia Horticulturae. 2017;219: 22-30. doi: https://doi.org/10.1016/j.scienta.2017.02.033.

36. Riofrío ML, Cruz D, Torres E, De la Cruz M, Iriondo JM, Suárez JP. Mycorrhizal preferences and fine spatial structure of the epiphytic orchid *Epidendrum rhopalostele*. American Journal of Botany. 2013;100: 2339-2348.

37. Peakall R, Ebert D, Poldy J, Barrow RA, Francke W, Bower CC, et al. Pollinator specificity, floral odour chemistry and the phylogeny of Australian sexually deceptive Chiloglottis orchids: implications for pollinator-driven speciation. New Phytologist. 2010;188: 437-450.

38. Phillips RD, Bohman B, Anthony JM, Krauss SL, Dixon KW, Peakall R. Mismatch in the distribution of floral ecotypes and pollinators: insights into the evolution of sexually deceptive orchids. J Evol Biol. 2015;28: 601-612. doi: https://doi.org/10.1111/jeb.12593.

S1. Table 1. Genetic diversity estimation for each population of two species (P. lilijae, M. rosea). N: number of individuals located in each population, n: number of individuals included in genetic analysis, P: percentage of polymorphic loci, I: Shannon's information index, He: expected heterozygosity.

Fig. 1

Click here to access/download;Figure;fig.1.tif

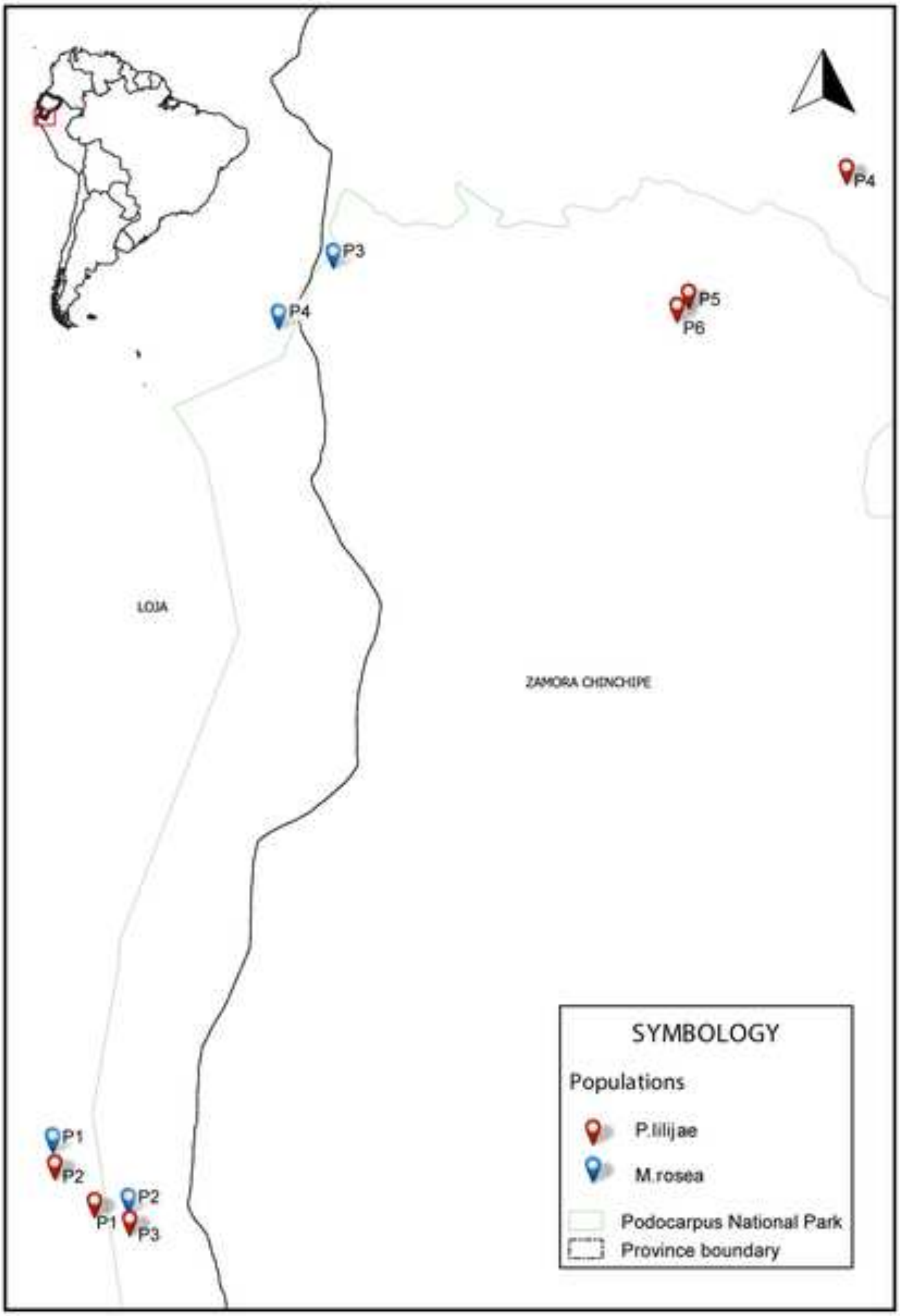

Fig. 2a

Click here to access/download;Figure;fig.2a.tif

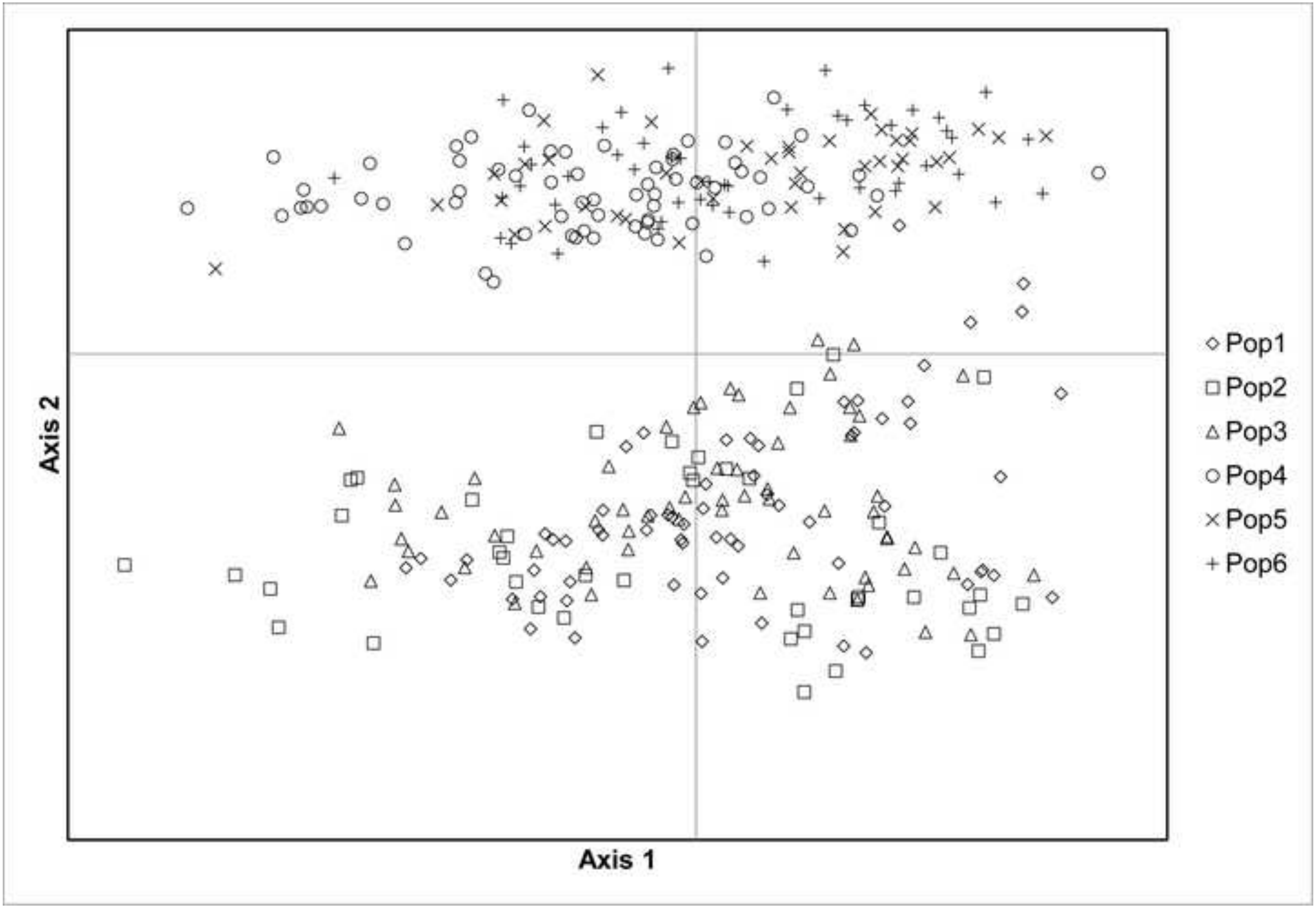

Fig. 2b

Click here to access/download;Figure;fig.2b.tif

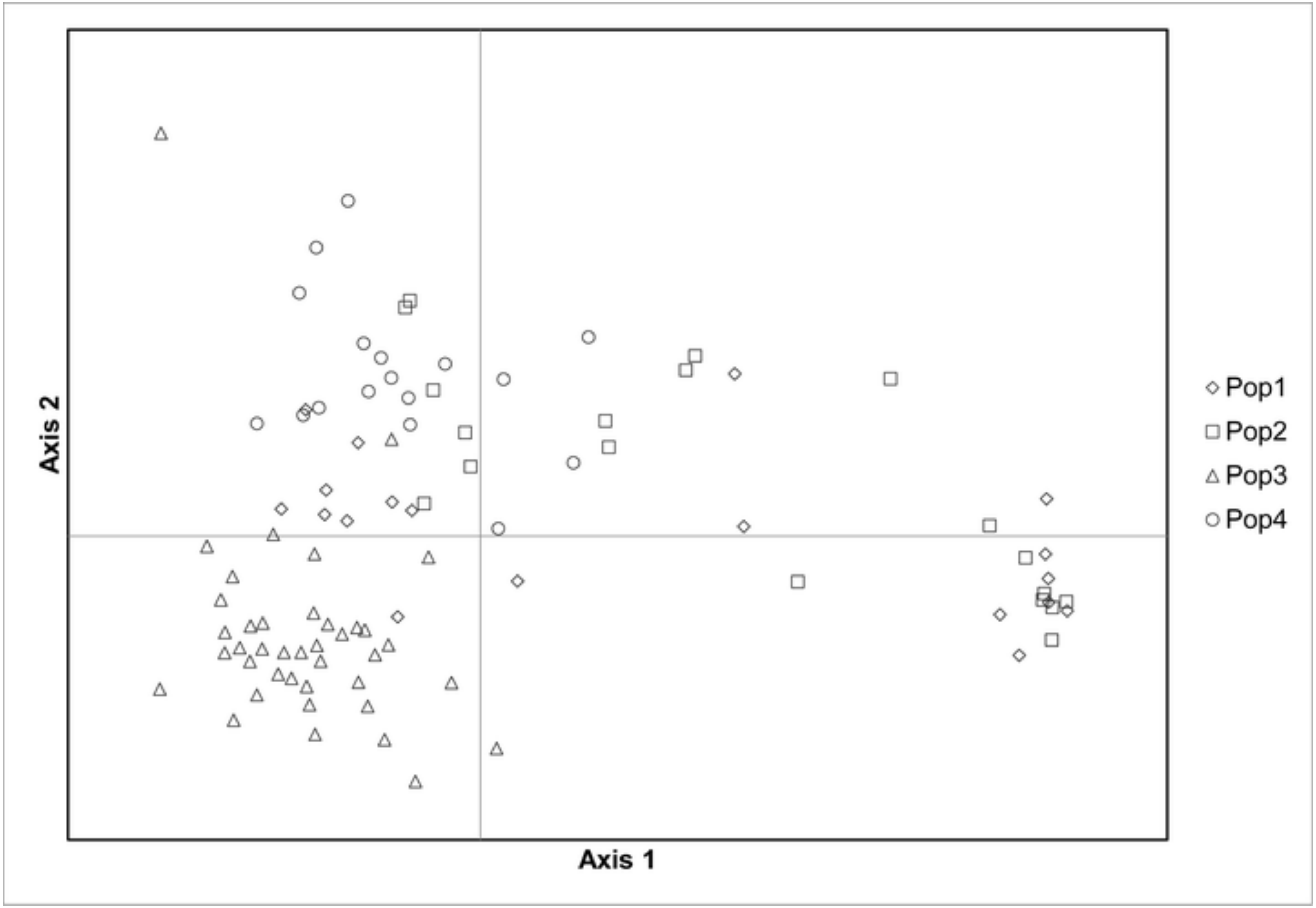

Fig. 3a

Click here to access/download;Figure;fig.3a.tif

a)

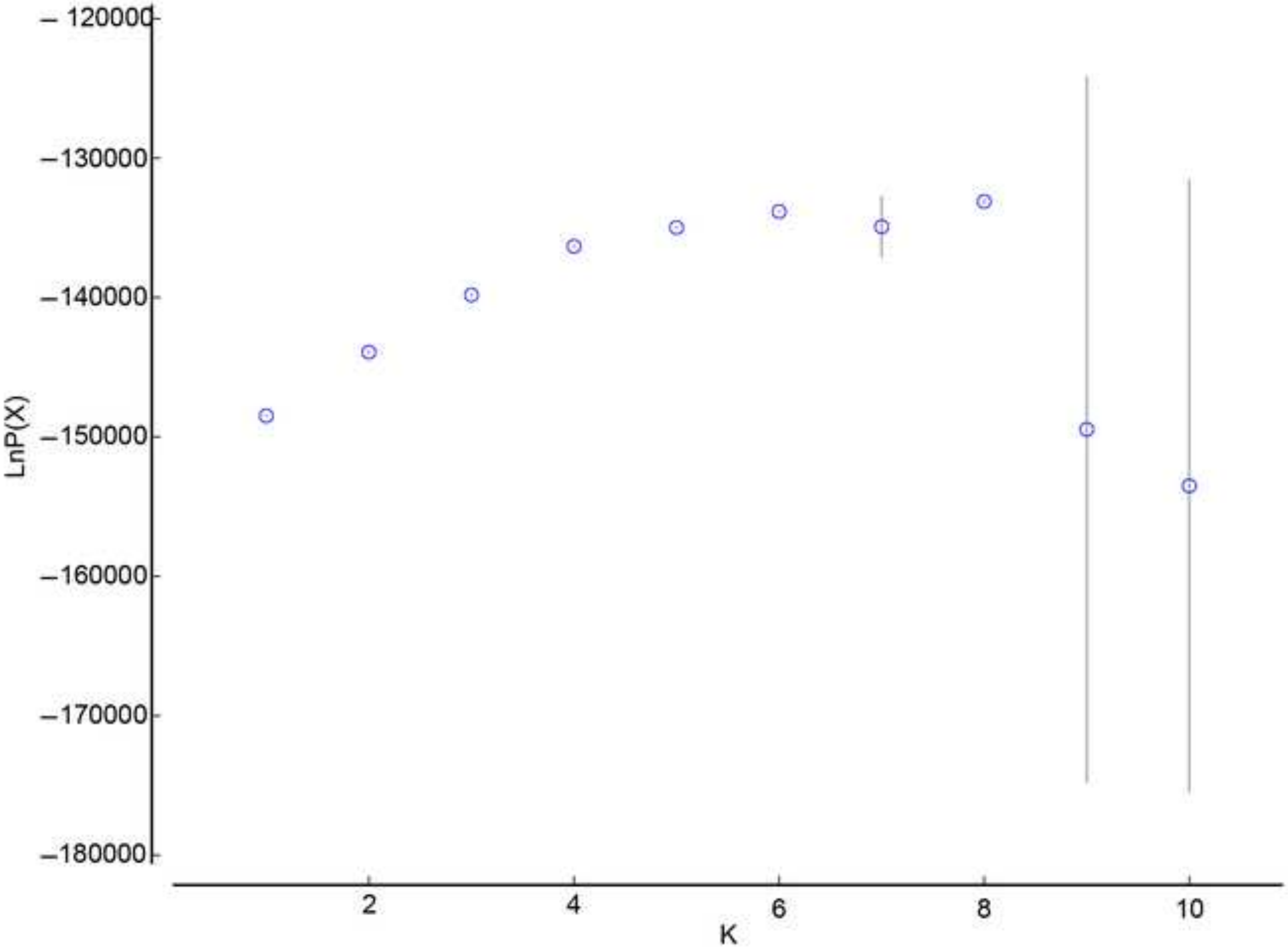

Fig. 3b

Click here to access/download;Figure;fig.3b.tif

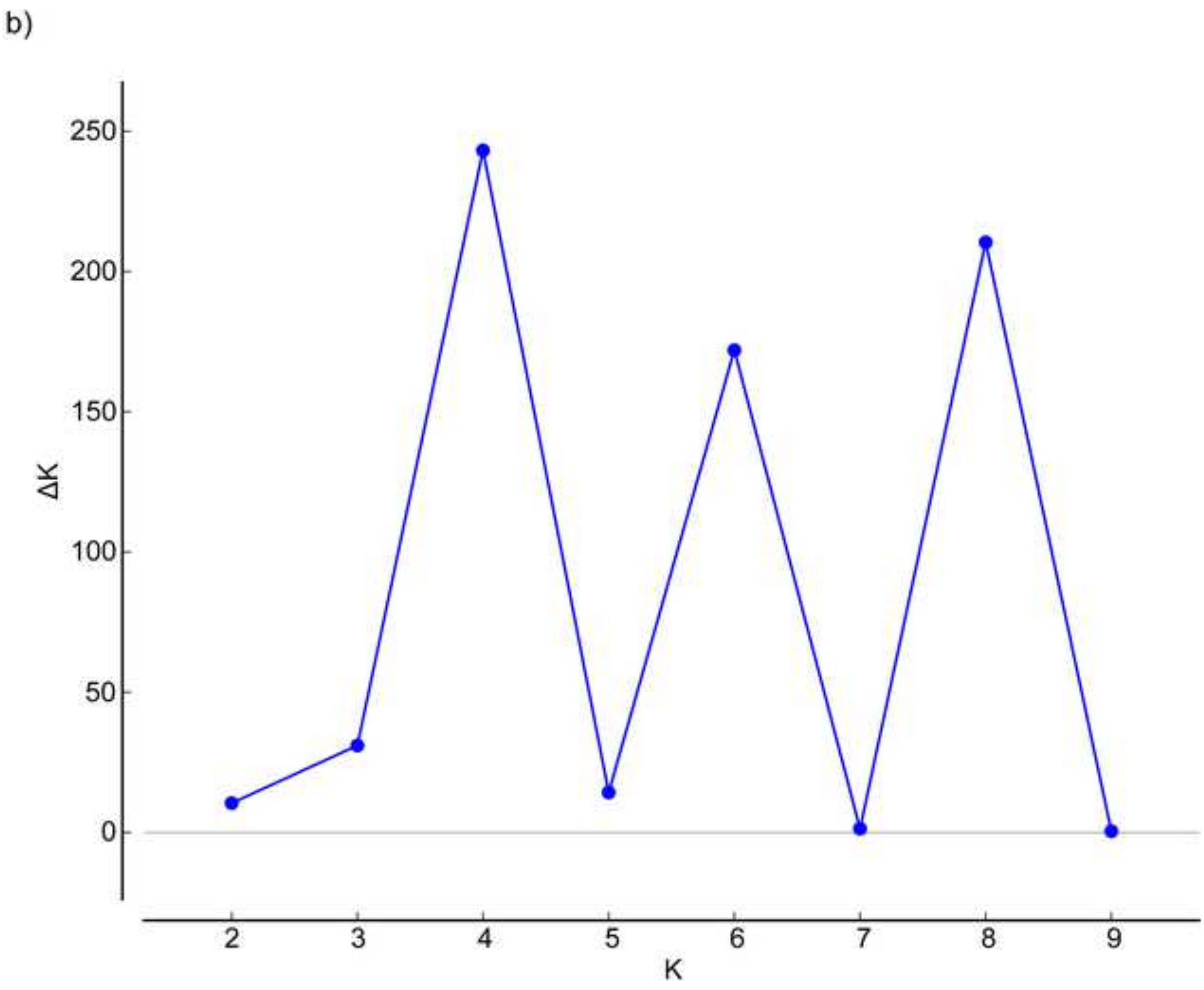

Fig. 3c

Click here to access/download;Figure;fig.3c.tif

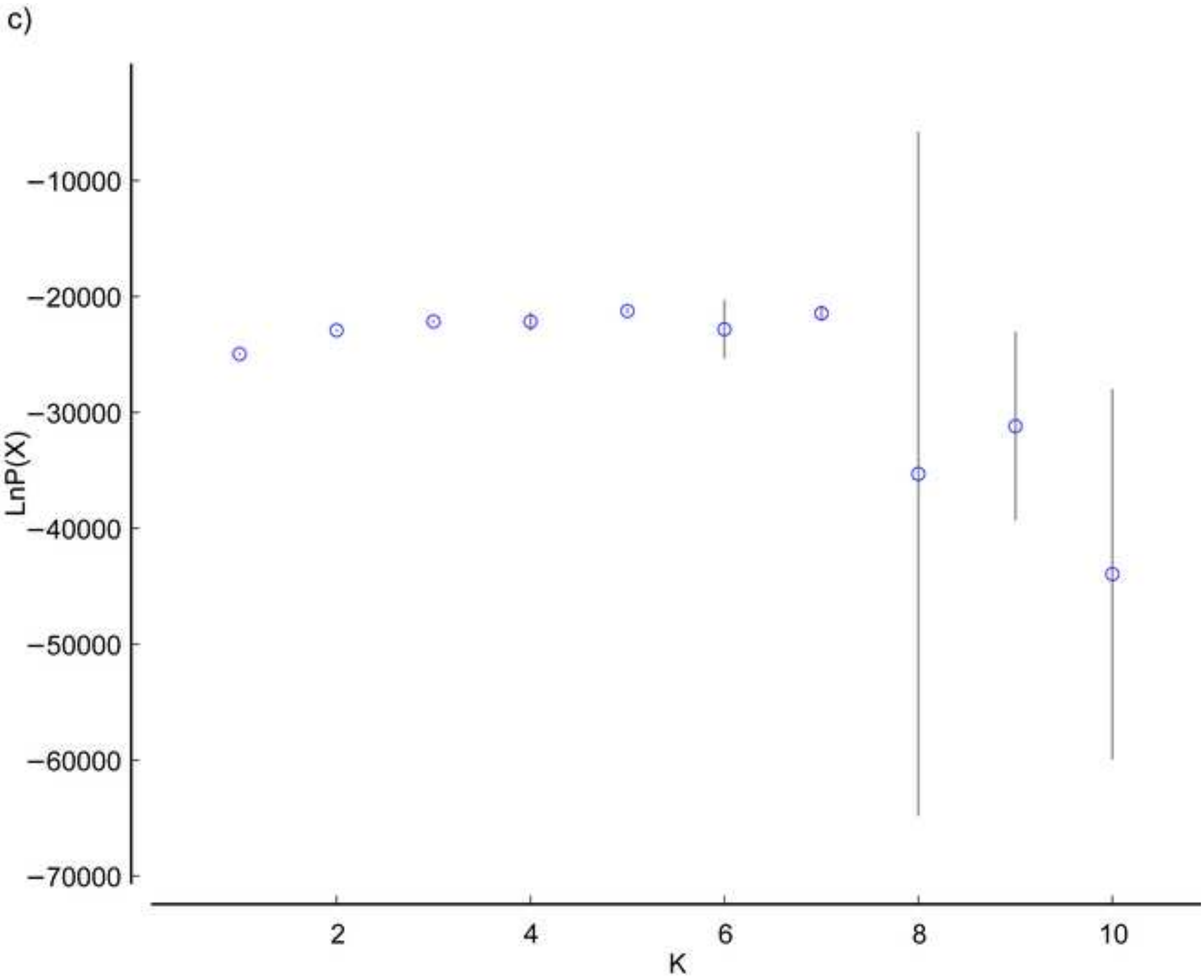

Fig. 3d

Click here to access/download;Figure;fig.3d.tif

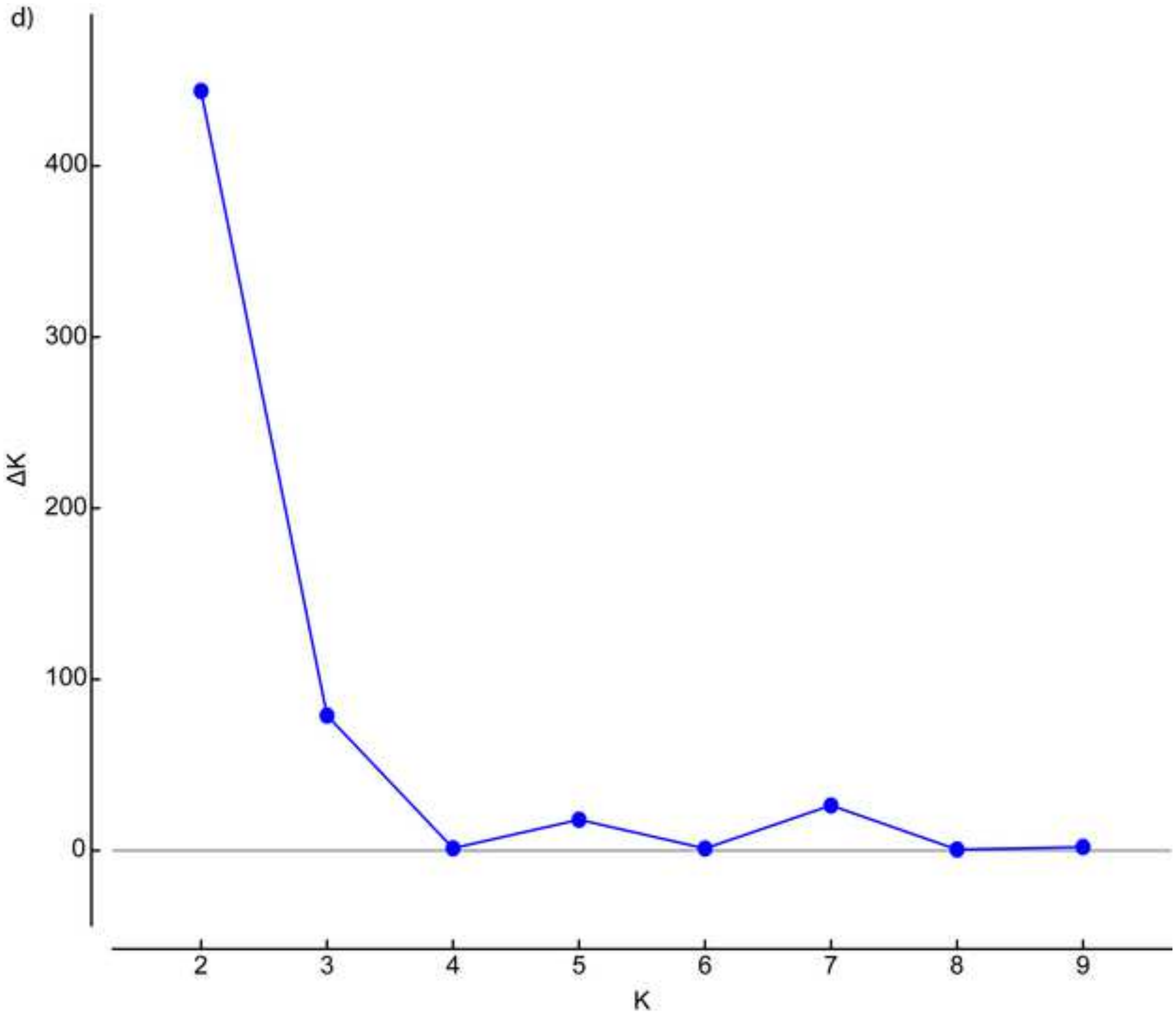

Fig. 4a

Click here to access/download;Figure;fig.4a.tif

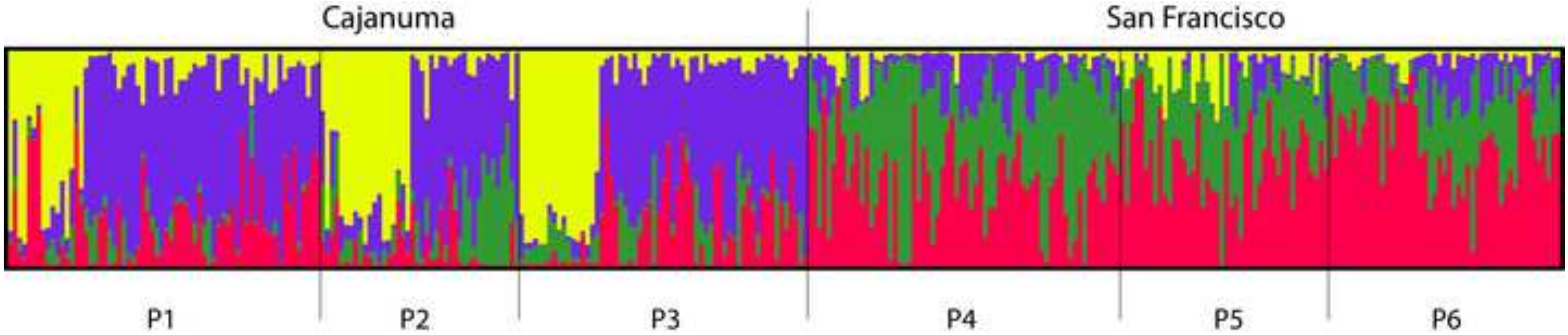

Fig. 4b

Click here to access/download;Figure;fig.4b.tif

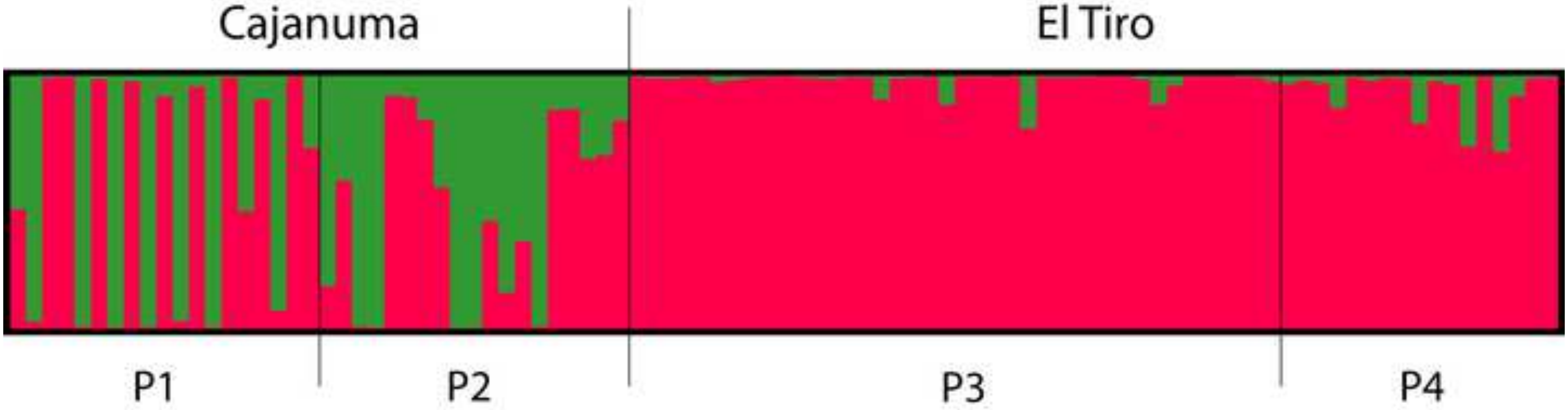

Fig. 5a

Click here to access/download;Figure;fig.5a.tif

a)

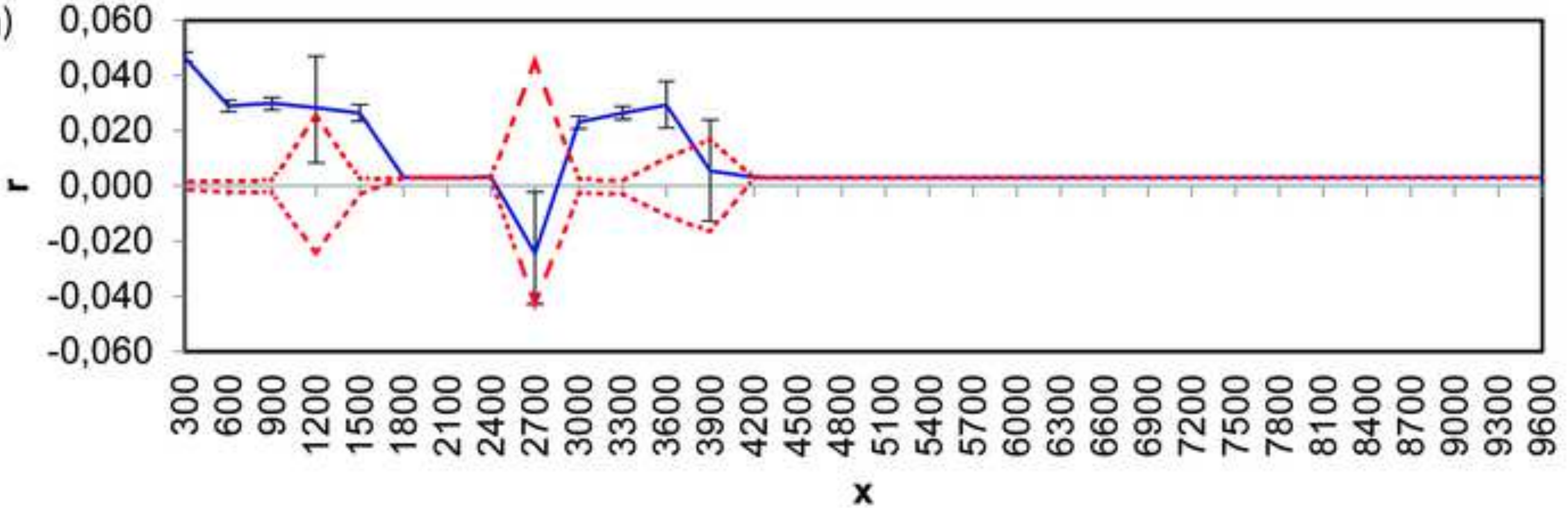

Fig. 5b

Click here to access/download;Figure;fig.5b.tif

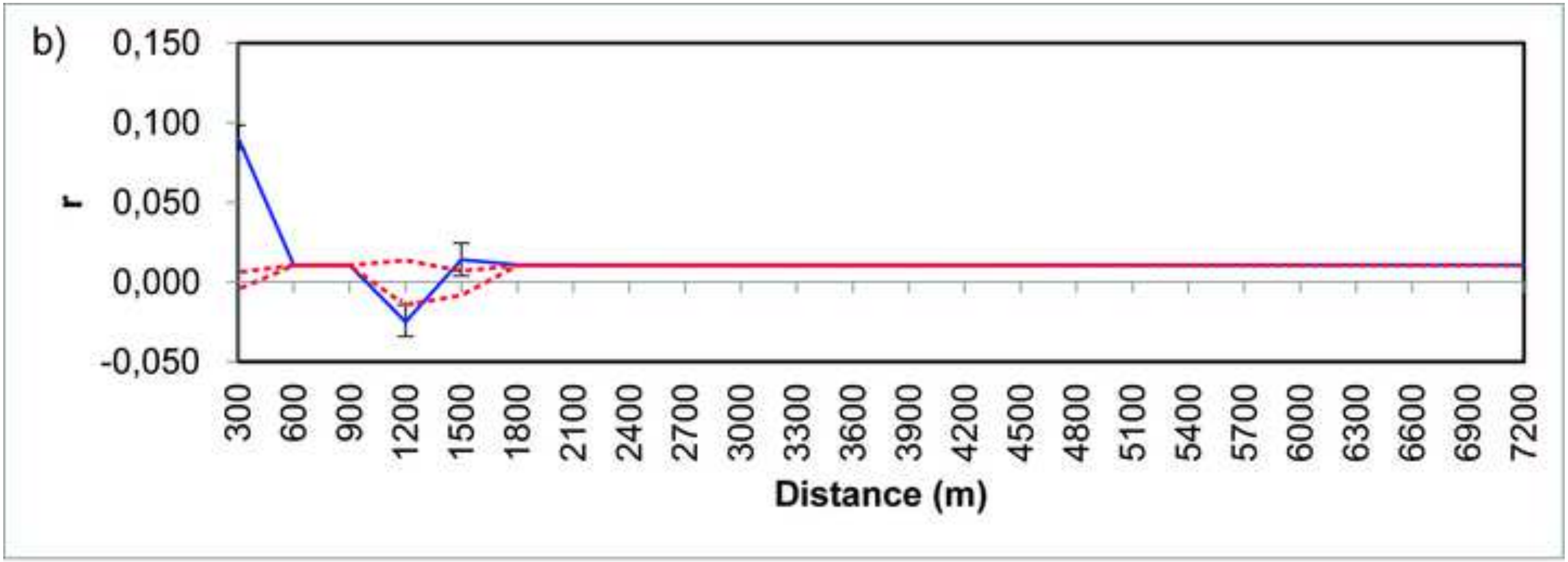

Fig. 5c

Click here to access/download;Figure;fig.5c.tif

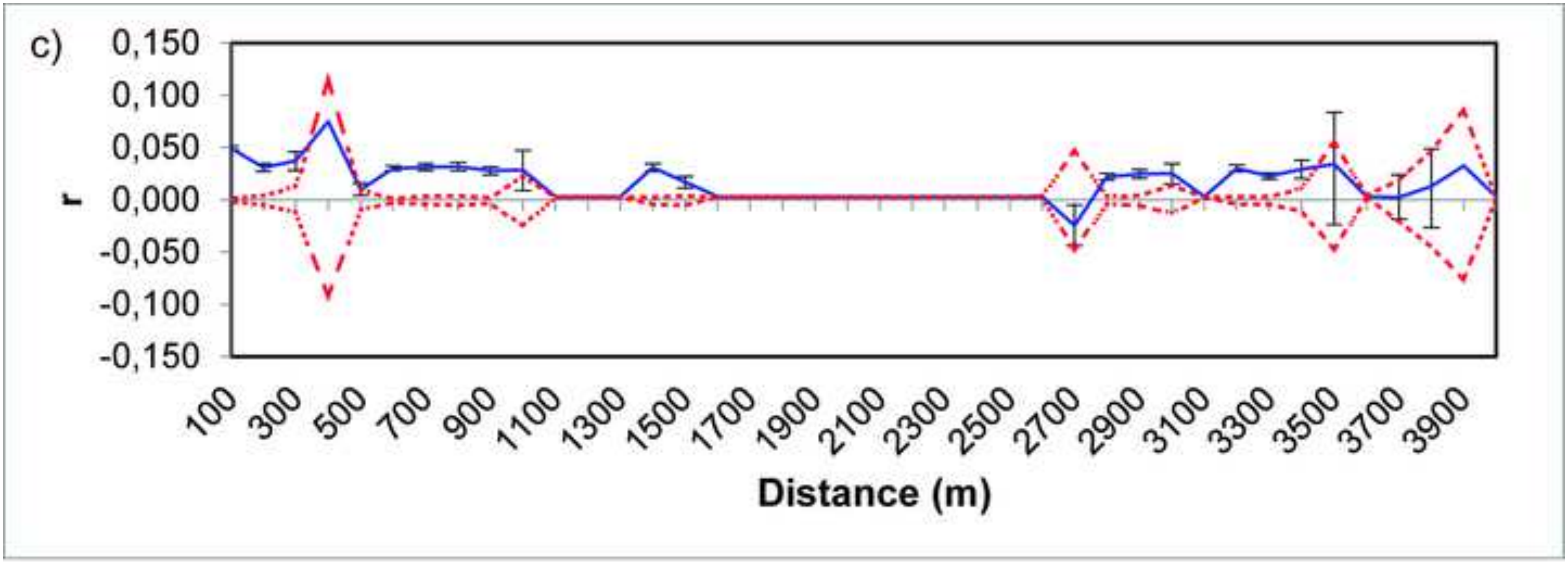

Fig. 5d

Click here to access/download;Figure;fig.5d.tif

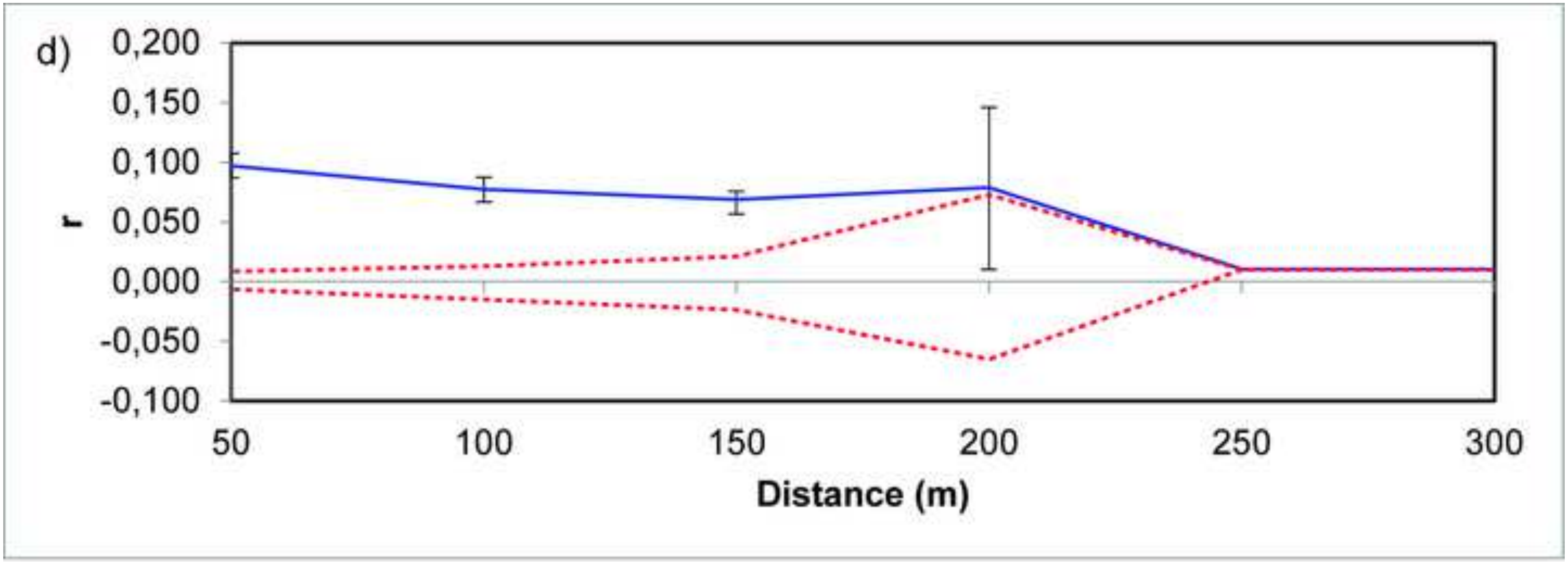

Click here to access/download
Supporting Information
S1. Table1.docx
